# Work Engagement in the Context of Confucian Culture: A Case of Chinese Civil Servants

**DOI:** 10.3389/fpsyg.2020.573146

**Published:** 2020-12-21

**Authors:** Xiaojun Lyu

**Affiliations:** School of International and Public Affairs, Shanghai Jiao Tong University, Shanghai, China

**Keywords:** work engagement, dynamic mechanism, grounded theory, civil servants, Confucianism

## Abstract

Although work engagement as a positive organizational behavior has gained considerable achievements in recent years, there is still a lack of content research based on certain culture, job, and group characteristics. This study conducts a grounded theory research on work engagement by coding and analyzing the interview files from public servants working in the government located in Eastern China. The result shows a five-dimension construct of work engagement, which includes loyalty to the work, commitment to the working relationships, positive emotion, initiative action, and priority for mixed roles. Additionally, the current study also constructs a theoretical model which discovers the dynamic variables motivating the process of work engagement and the influence of Confucianism.

## Introduction

With the continuous evolution of organizational environments and tasks, workplaces tend to be psychologized—that is, employees need to have sufficient psychological adaptability and engagement level to complete organizational tasks. As a typical manifestation of positive organizational behaviors, work engagement has received much attention in academia. Using the keyword “work engagement,” related studies were retrieved from SSCI journals. Over the past 5 years, the number of published articles on work engagement has increased from 142 (2014) to 224 (2019), showing an obvious uptrend. Work engagement reflects individuals’ positive and spirited mentality. Studies have proved the close relationship between work engagement and employee loyalty (Vigoda-Gadot et al., 2013), subjective career success (Muser et al., 2012), and work performance (Bakker and Bal, 2011).

In China, the concept of work engagement has maintained different meanings. On one hand, engagement is perceived as career value which acts as a norm for professional behaviors. On the other hand, most of academic research on work engagement follows the theoretical framework developed in the Western culture. However, work engagement, from its nature, is definitely connected with the workplace and the underlying culture characteristic. Therefore, it is necessary to rethink the construct of work engagement in a specific culture context. Chinese Confucian culture is undoubtedly different from the Western culture, which must have effects on people’s behaviors in the workplace. Additionally, compared with private organizations, work requirements in public sectors have their own characteristic. To motivate the expected behaviors of civil servants depends on whether the construct of work engagement has been discerned and measured correctly.

The current study will start from the literature review, and then develop the construct of work engagement based on the group of Chinese civil servants following the paradigm of grounded theory. The research result will have its theoretical contribution to the domain of work engagement in several ways. Firstly, there is very little research linking work engagement and civil servants’ motivation and performance. We do not have much knowledge of specific work engagement concept under the background of public sectors till now. As such, it is an urgent task to explore the connotation and structural boundary of civil servants’ work engagement, which will enlarge the theoretical picture in this domain. Secondly, previous construct of work engagement might focus on the employee’s psychological states or behaviors in the workplace. Whereas, the current study is going to explore the content of engagement from a situation-based perspective. If the dimensions of work engagement and its antecedent variables can be explored clearly, it would act as an instrument of performance management. The main purpose of current study intends to address a comprehensive model of work engagement including the dynamic variables motivating people involve into their work. Furthermore, as stated above, the positive working behaviors are connected with their surroundings and probably shaped by job requirement or organizational culture. In the process of socialization, individuals’ cognition and behaviors are unconsciously influenced and shaped by culture norms. Organizational socialization directs employees’ behaviors using training or rewarding systems. Consequently, an abstract concept independent of specific working and culture situation has less value for us to know the characteristic of work engagement. To our knowledge, previous frameworks of work engagement are usually based upon participants from Western culture. Hofstede (1993) improved the culture difference between Chinese sample and western counterparts, especially in terms of Confucian dynamism. Culture modifies the values and behaviors people hold and engender thoughts and actions in their lives. For example, individualism emphasizes one’s independence and self-achievement, while Confucianism weights interdependence more and behaviors conforming to other’s expectation are more socially desirable (Triandis, 1995). Comparatively, considering the background of Confucianism, there has little research about the characteristic of people’s involvement and dedication in their work. Thus, this study is going to fit the gap by developing a framework of engagement embedded in Confucian culture.

## Literature Review on Work Engagement

Initially, work engagement represented a combination of employees’ selves and work roles, as well as a dynamic dialectical relationship between individuals and work—on the one hand, people devote physical and mental efforts to work, and on the other, work roles provide them with the possibility of self-expression, satisfying the need to fulfill their working selves (Kahn, 1990). Work engagement is considered to be a state of utter engagement in work, and hence, early studies placed engagement and burnout at two ends of a continuum—the burnout state (e.g., emotional exhaustion, cynicism, and inefficacy) at the negative end and the engagement state (e.g., spiritedness, utter devotion, and efficacy) at the positive end; people with a higher level of work engagement are unlikely to feel worn out, and vice versa (Maslach and Leiter, 2008). Although engagement is closely linked with burnout, the studies pointed out that the two concepts at opposite ends do not correspond to each other (Kim et al., 2009). Subsequent studies have tended to consider engagement as an independent concept—to be more specific, work engagement represents a positive and spirited mentality and is characterized by vitality, devotion, and concentration. The Utrecht Work Engagement Scale (UWES), which was designed based on this theory, has been widely applied in quantitative studies of engagement (Schaufeli and Bakker, 2010). The concept of engagement contains emotional elements; inspired by emotional traits and states, researchers argued that engagement may also be a state of work engagement. The experience sampling method shows that an individual’s level of engagement fluctuates with events or experiences from day to day or week to week (Ouweneel et al., 2012). Log studies have shown that differences between individuals account for 58% of variation in engagement, whereas internal changes within individuals account for 42% of it (Volman et al., 2013).

In China, “respect” plays a fundamental role in traditional Confucianism and covers diverse aspects, including respect for heaven, for people, for things, and for work (i.e., work engagement). The core of work engagement in traditional culture manifests in “loyalty.” *Yi Zhuan* (an ancient classic work) contends that loyalty and honesty are the pathways to improving moral cultivation and academic achievements, and respect for work (i.e., work engagement) is to be loyal to work without distractions. *The Combined Collected Works of the Ice-Drinking Studio* (written by Liang Qichao) argues that whatever you choose to do, you must complete it perfectly. To achieve this goal, the only solution is loyalty—that is, do it devotedly (Fang, 2017).

In the field of organizational management, Chinese studies have explored the concept and construct of work engagement across different occupational groups. A survey of 191 government functionaries showed that the engagement of Chinese government functionaries can be measured in two dimensions, namely devotion and vitality (Yang and Liao, 2011). Using a self-developed evaluation for work engagement, a test was conducted on employees of six enterprises, and the results showed that work engagement comprised six factors, which were task focus, vitality, active participation, value introjections, efficacy, and active support (Zeng and Zhao, 2009). Among primary and secondary school teachers, the results of an interview and questionnaire survey showed that work engagement had six dimensions, namely dutifulness, conscientiousness, diligence, cognitive engagement, selfless devotion, and innovativeness, and among college teachers, the results of factor analysis showed that work engagement had three dimensions, which were work involvement, organizational identification, and active participation (Guo, 2012). Though the above studies have discussed work engagement in the context of Confucian culture and in working scenarios, they did not reach any consensus, or construct a conceptual structure with the appropriateness of cultural scenarios.

Therefore, in keeping with grounded theory, this study will explore the content of work engagement and its dynamic drivers, which is expected to give us a better understanding of how engagement operates in a Confucian setting.

## Research Design

### Methodology

This study adopted the grounded theory approach, which is a method for qualitative studies. This approach is for developing substantive theories (i.e., theories that are applicable in a specific spatiotemporal context) that range between the grand ones and those with micro-operational value, not excluding universal formal theories (Chen, 2015). The approach follows this operation path: (1) collect data through theoretical sampling, and then encode it to generate subcategories; (2) analyze the relationship between the data and concepts; (3) repeat the above steps to ensure theoretical saturation; and (4) improve the relationship between subcategories and perform theoretical integration (Kathy, 2020). Data collection and coding are core tasks in grounded theory—data should be collected so as to ensure theoretical opening and sensitivity. Data coding comprises open coding, axial coding, and selective coding (Juliet and Strauss, 2017). The grounded theory approach is rooted in phenomena, highlights the characteristics of scenes, and extracts the core concepts and categories of the original data in the process of repeated comparison, analysis, and refinement. Therefore, it is appropriate to construct a theory concerning work engagement in this study.

### Sampling and Participants Characteristic

This study employed theoretical sampling, which is a concept-based or theme-based method of data collection. Its aim is to collect data from real scenarios (e.g., places, people, and events), develop concepts maximally from attributes and dimensions, explain the connotations of concepts, and identify the relationship between them (Liu and Li, 2017). The process of theoretical sampling comprised three stages: (1) interviewing 37 respondents of a district-level functionary training project of a city in East China in the first stage (March to April 2018); (2) interviewing 10 respondents of a university Master of Public Administration (MPA) course project in the second stage (May to June 2018); and (3) interviewing five interviewees working in the public sectors in the third stage (November to December 2018), to ensure sample saturation (newly added data cannot provide new information, subcategories, or themes). Sample saturation was ensured through data analysis, comparison, and sample replenishment in these stages. Thus, in this study, a total of 52 interviews were conducted. Table 1 describes the sample characteristics.

**TABLE 1:** Sample characteristics.

Characteristics	Group	*N*	Characteristics	Group	*N*
Sex	Male	19	Education background	Bachelor’s degree or below	39
	Female	33		Master’s degree	13
Age (M)		27.12	Tenue (M)		5.39

### Design of Interview Framework

A depth interview is a means to collect and analyze data and allows researchers to communicate with interviewees through a semi-structured interview framework. This framework leaves room for the open-ended questions and expressions. Therefore, researchers may refocus the questions and ask follow-up issues according to interviewees’ statements, thus capturing in-depth information and obtaining more extensive and substantial original data.

According to the objectives of this study, the interview framework focuses on two core questions: (1) What do you think are the manifestations of work engagement and burnout? and (2) Can you put forward to specific examples of work engagement? During the interview process, the researchers paid special attention to the following interviewee information: (1) events (what specific things have happened in the engagement events or burnout events?); (2) actions (what specific behaviors did the person have when he/she is engaged or burnout in those events?); (3) motivation (why did the person do so?); (4) results (what results did the actions cause?); and (5) environment (in what circumstances would the person do so?). The duration of each interview ranged from 10 to 20 min. The researchers prepared interview records on a word-by-word basis and handed them over to the interviewees to check for errors between their statements and the records.

### Data Processing

During the interview process, the researchers also encoded the collected interview data. Using the NVivo11 qualitative analysis software, text content was coded on a word-by-word basis. The NVivo11 can clearly record the steps of analysis, and archive the results of each, consequently, presenting the whole research process well and ensuring the rigor of the study (Liu and Li, 2017).

## Data Analysis

Based on the classical procedure of constructing grounded theory, this study used a three-level coding process—open coding, axial coding, and selective coding.

### Open Coding

Open coding follows this path: (1) extract concepts from original data; (2) compare new data with the extracted concepts continuously in the process of extraction and formation of concepts; and (3) explore the relationship between data and concepts. Coping intent is to identify new attributes of concepts, extract new concepts, or change the terminological expression of concepts. Open coding is also considered fresh coding, during which concepts are preferably named in terms of interviewees’ words rather than the researchers’. Through a descriptive generalization of original data, the researcher extracted 93 concepts. Their total sample size reached 396. The concepts were preliminarily processed and summarized according to the internal logical relationship between them. In particular, concepts with similar or interrelated meanings were further grouped into 15 subcategories (Table 2).

**TABLE 2:** Concepts and subcategories developed through open coding (excerpted sample).

Number	Subcategory	Number of node samples	Concept
1	Expertise in work	41	(1) Know the contents of the work fully; (2) understand the work from a unique perspective; (3) have a good understanding of social conditions and public opinions; (4) be familiar with departmental business and related laws, regulations, and policies; (5) watch Internet TV programs through the work platform (−); (6) wash vegetables and cook in the office (−).
2	Identification with work	18	(1) Be fascinated with the work and consider it a pleasure; (2) have a strong sense of identification with organizational value and take pride in it; (3) treat the work respectfully and seriously; (4) regard the work as an important undertaking; (5) consider overtime not worthwhile (–); (6) no consistency between personal value and organizational value (–).

### Axial Coding

Based on open coding, axial coding is for developing categories and dimensions, define their attributes, and try to develop the relationship between different categories (Juliet and Strauss, 2017). Table 2 shows that the 15 subcategories are mainly linked in two ways: similar meanings and a causal relationship. For subcategories with similar meanings or attributes, the researcher may further define their meanings and highlight the characteristics of their different dimensions as primary categories. For example, “expertise in work” and “exploratory spirit in work” can be refined as “respect for work”; “commitment to service objects” and “mutual commitment with colleagues” can be refined as “relationship commitment”; and “priority to work affairs” and “handling the relationship between personal gains or losses and collective needs” can be refined as “priority in trade-off of conflict.” The causal relationship mainly manifests in two aspects: (1) some subcategories (e.g., “identification with work,” “money,” and “promotion”) are motivations for increased work engagement, and (2) a series of states and degrees of work engagement lead to “work performance.” Table 3 describes the primary categories developed through axial coding, as well as their meanings.

**TABLE 3:** Categories developed through axial coding and their meanings.

No.	Category	Subcategory	Meaning
1	Respect for work	(1) Expertise in work (6) Exploratory spirit in work	(1) Have an accurate and in-depth understanding of the work; (2) pursue excellence in accomplishing tasks; (3) show respect for the work, and not act arbitrarily.
2	Relationship commitment	(3) Commitment to service objects (4) Mutual commitment with colleagues	Know the behavioral roles in the interactive relationship, fully consider the needs of relational objects, and maintain effective cooperation with them.
3	Initiative in the behavioral process	(5) Degree of work engagement	Be engaged in work actively, envisage difficulties bravely, and do not shirk responsibility.
4	Emotional fullness	(9) Emotional state in work	Have positive emotions toward the work, be emotionally stable in work, be enthusiastic about people and things, and do not complain.
5	Priority in trade-off of conflict	(7) Priority to work affairs (8) Handling the relationship between personal gains or losses and collective needs	Give priority to work and emphasize collective interests when dealing with the conflicts of interest between different roles.
6	Internal motivation for work engagement	(1) Identification with work (11) Satisfying psychological needs	Be engaged in the work actively out of love for the work itself and for satisfying inner psychological needs.
7	External motivation for work engagement	(12) Money (13) Leadership (14) Promotion (15) Organizational environment	Active work engagement is mainly motivated by the rationality in salary, leadership, promotion system, and allocation of other organizational environmental resources.
8	Performance	(10) Work performance	Effectiveness and results of task accomplishment.
			

### Selective Coding

**FIGURE 1:**
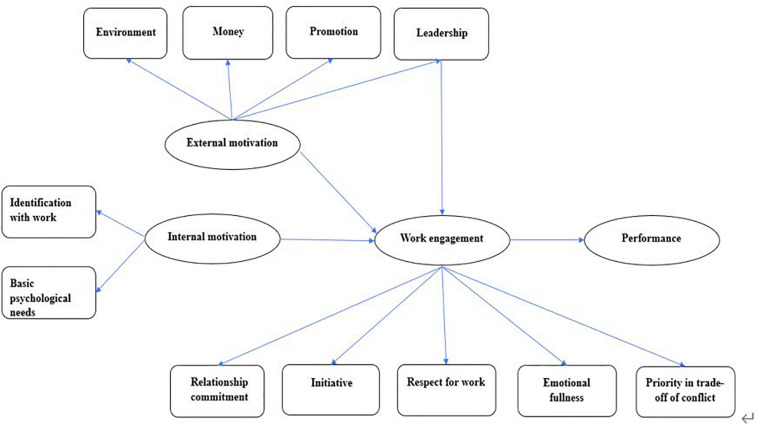
Theoretical model for structure and motivation of work engagement.

The main aim of selective coding is integration, or to be more specific, linking a core category with other categories, and extracting and developing corresponding theoretical constructs. The core category is the central concept that can integrate all other related categories (Juliet and Strauss, 2017). The results of axial coding in Table 3 were analyzed in great depth. The researcher was of the opinion that respect for work reflects the cognition of work; relationship commitment, “emotional fullness,” and “initiative in the behavioral process” reflect the behavioral expression and emotional state in work; and “priority in trade-off of conflict” reflects a work-first value orientation. The core attributes of these categories manifest as a positive working state and integrate a series of psychological and behavioral characteristics of work-related cognitions, emotions, actions, and value orientations. Thus, this study argues that work engagement can, as the core category, govern the above categories. According to the coding paradigm proposed by Straus (i.e., condition − action strategy − result), different subcategories are linked in the ordinary order of development of things (Strauss and Corbin, 1990). The motivations for positive working states usually include both internal and external motivations. In this study, internal motivations are identification with work and “satisfying psychological needs,” and external motivations money, “leadership,” promotion, and “organizational environment.” The results of a positive working state manifest as effective work performance. According to the logical relationship between categories, this study presents a theoretical model for the structure and motivation of work engagement (Figure 1).

### Reliability and Validity Test

It is difficult to evaluate the quality of grounded theory studies (Seale, 2002). In this study, a self-test was performed in terms of the four indices proposed by Charmaz—reliability, originality, resonance, and effectiveness. Reliability indicates whether the subcategories developed through coding cover the information of empirical observations, and whether there is a strong logical relationship between the collected data and their analysis. In this study, continuous comparison coding was employed. The whole interview and coding process lasted 10 months; data collection was concurrent with coding, until no new subcategories were available, to ensure that the subcategories cover the desired information. In addition, internal consistency was used as a quantitative index of reliability. Two encoders (a professional researcher and a master’s degree candidate in the same discipline) were invited to participate in postcoding, and they shared a consistent understanding of the study theme. The reliability coefficient was equal to the ratio of the number of samples with consistent coding for each subcategory. The calculation equation is CI = 2 × *S*/(*A*_1_ + *A*_2_) × 100%, in which *S* denotes the number of samples with consistent coding, and *A*_1_ and *A*_2_, respectively, denote the number of samples coded by the two encoders. For example, the coding consistency index of Subcategory 1 is CI = 2 × 31/(39 + 43) × 100% = 0.77. The coding consistency indices of the 15 subcategories range from 0.64 to 0.87, meeting the specified requirements.

Originality indicates whether the extracted subcategories and categories are fresh, and whether they provide new insights into the study objectives. As mentioned above, studies of work engagement have been carried out for several years, but studies based on China’s grass-roots public sectors are really few. This five-dimension work engagement construct extracted in the current study is significantly different from traditional ones.

Resonance indicates whether subcategories fully describe the respondents’ experience. After the completion of data coding, two civil servants from public sectors were invited to read the concept definitions of subcategories and categories. They unanimously agreed that the names and meanings of those five dimensions were in line with real working environments. Additionally, the feedback from two civil servants shows the current study has its effectiveness to provide people with an interpretation of the daily world.

## Conceptual Interpretation and Model Developing for Work Engagement

**FIGURE 2:**
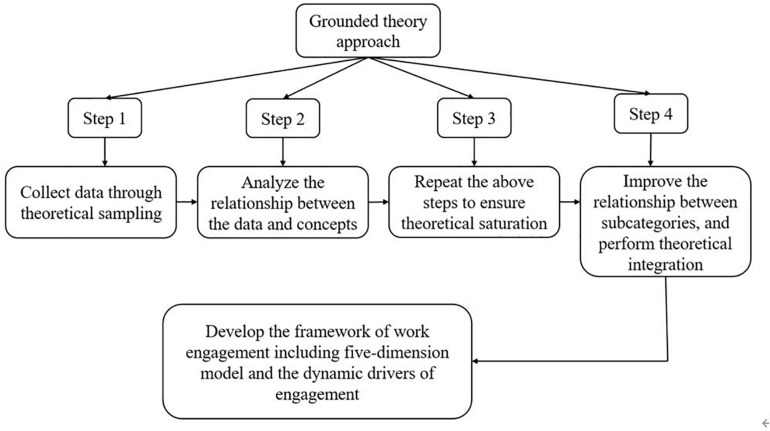
Research steps and results.

Using the grounded theory approach, this study identified the meaning and construct of work engagement and helped people understand its specific characteristics and potential motivations, thus facilitating related evaluation or management activities in practice. Figure 2 shows the methodology steps and the results.

### Construct of Work Engagement

In the previous studies of work engagement, several theoretical frameworks have been developed. In Kahn (1992) model, engagement was described as a motivational concept including physical, emotional, and cognitive energy. Macey and Schneider (2008) considered work engagement a comprehensive taxonomy which contained not only personality and affect (trait engagement) but also psychological empowerment (state engagement) and proactive behavior (behavioral engagement). The most well-known model comes from Schaufeli et al.’s (2002), work engagement was defined as a positive state of mind characterized by vigor, dedication, and absorption. These studies carry rich connotation of work engagement, which focus on personality traits, psychological states, attitude, and behavior typologies. However, these frameworks show their abstract and universal knowledge in this domain, instead of a more specific perspective to understand persons’ engagement in their working surroundings.

Work engagement reflects the relationship between people and work; in particular, people acquire a sense of self-identity through work, and the work in turn becomes a carrier to satisfy their needs. The way to allocate people’s resource between work and other life activities shows their priority identity. Consequently, it is necessary to demonstrate the working situations including tasks, relationships, as well as conflicts people have to manage in their work. The characteristics of people’s reactions in dealing with those situations indicate their levels of work engagement.

Using the grounded theory approach, this study developed an integrated construct of work engagement. Compared with previous studies, this study has the following characteristics:

First, it discusses the mental and behavioral states of integrated work engagement. It identifies five dimensions in the engagement construct: respect for work, emotional fullness, initiative in the behavioral process, relationship commitment, and priority in trade-off of conflict. The first three reflect the cognitive, emotional, and behavioral characteristics in the process of work engagement. These three dimensions not only include the vitality and emotional states of the previous engagement concept but also reveal the concrete manifestation of enthusiastic work engagement, helping people acquire a more in-depth understanding of it. The fourth dimension reflects the attitude to work relationships. Most work activities involve maintaining relationships. The participants of the current study are from public sectors, and one of their work objectives is to gain people’s trust. In addition, task collaboration in the process of public services is also indispensable to their work. Therefore, work engagement must encompass commitments to service relationships, as well as the colleague relationships. The last dimension pertains to value trade-off—that is, priority in resource allocation in case of role conflict. Studies have shown that people’s work engagement is mainly due to the reason that the work would satisfy their basic psychological needs; work thus becomes an important scenario for realizing self-concepts (Kahn, 1990). The priority in trade-off of conflict represents the choice made by the individuals. The stable tendency to the work issues shows a higher level of work engagement.

Second, the concept of work engagement specified in this study presents Chinese traditional cultural characteristics. In the ideological system of Confucianism, *The Works of Zhu Zi* points out that people with a respect for work can concentrate on their work. This paints a vivid portrait of such people—that is, them being absorbed in whatever they choose to do. To be successful in something, people must be absorbed in it, and try to improve their techniques and mentality (*Xun Zi: On Military Strategy*). In this study, respect for work reflects an exploratory spirit and pursuit of excellence in work. In addition, Confucianism advocates “faithfulness to duties,” which is the basic principle of engagement; people are faithful as long as they do their best, make efforts, or behave themselves (Liu and Xiao, 2016). In other words, faithfulness to duties manifests through the fact that people make every effort in work or are considerate toward others wholeheartedly. In this study, the cognition of civil servants for work engagement reflects their respect for and commitment to the work they are engaged in and the purposes they serve. Initiative in the behavioral process and emotional fullness indicate that people make every effort and devote everything (e.g., emotions and action resources) to work. In addition, the Confucian outlook on justice and benefit is also verified in the current concept of work engagement. “Justice” usually refers to the code of ethics in society and “benefit” to material benefits or utilities. Regarding the relationship between them, Confucianism argues that justice outweighs benefits; in particular, people can experience enjoyment and spiritual satisfaction from a moral style of life (even if in great distress) (Yang, 2016). In the work engagement structure proposed in this study, “priority in trade-off of conflict” reflects the order of priority in the conflict between personal interests and work requirements. Employees with a high degree of work engagement usually give up personal interests or overcome personal discomfort while giving priority to work tasks.

### Model for Motivation Mechanism of Work Engagement

According to the concept of work engagement herein, this study argues that engagement is a process of making tendentious choices and allocating physical and mental resources based on the understanding of the internal and external environments. Using the grounded theory approach, this study also identifies the motivations for engagement. In keeping with the self-determination theory and conservation of resource theory, this study discusses the mechanism of these motivations.

The self-determination theory contends that individuals are positive organisms, and their perceptions and behaviors are a utility function that integrates social environments and internal resources. The key to maximizing the utility function is to satisfy three basic psychological needs—autonomy, competence, and belongingness (Van et al., 2016). Among the internal motivations identified in this study, satisfying psychological needs covers autonomy and feedback in work, a sense of achievement, and affirmation from others. It reflects the effects of autonomy and abilities on the behaviors of work engagement. Identification with work covers love for the work itself and value matching. It is a typical motivation with full autonomy. The external motivations identified in this study are organizational environment, promotion, leadership, and money. The content analysis of node samples shows that there are diverse organizational environment factors, including organizational climate, various norms, and even power relationships. Relatively speaking, the other three factors are more specific and well defined. From them, promotion had the largest number of node samples. A lot of coding content focuses on “reduction in work engagement due to limited and biased allocation of promotion resources.” Career promotion of public sectors is always considered the main constraint vis-à-vis work enthusiasm. Although the current incentive system has increased promotion channels, people’s attention to career promotion is nevertheless significant. In fact, this reveals that the promotion system reflects the basic need of people of gaining recognition for professional competences. In addition, leadership is a significant external motivation. Analysis shows that the effects of leadership on work engagement manifest in two aspects—on the one hand, leaders manipulate promotion activities, and on the other, their personality, charm, and supporting behaviors give employees a sense of belonging, thus facilitating work commitment.

The above findings have important policy implications with respect to incentive management of public sectors. First, it is necessary to further perfect the promotion system of public sectors in China. After the new Civil Servant Law of the PRC came into effect, the number of posts has been a bottleneck with respect to career development of the vast majority of grass-roots civil servants. In addition to performance and relationship bias, some inexplicable factors determine the results of career promotion (Ma et al., 2015). Therefore, the key of incentive system is to make improvements in these issues. Second, it is necessary to attach equal importance to rule by law and rule by virtue. While establishing new rules and regulations, leaders must also improve self-cultivation and self-discipline, which have a far-reaching influence on their subordinates’ work engagement.

The conservation of resource theory contends that people try to acquire, hold, and protect what they consider valuable, including material, social, personal, and vigor resources. Individuals who initially have abundant resources have an advantage and are willing to participate in activities to obtain more valuable resources. In contrast, individuals deficient in resources tend to exhibit more defensive behaviors to avoid the loss of the existing resources. The motivation variables identified in this study all have resource attributes. Internal motivations fall under individual resources, and external motivations are organizational environment resources, supporting resources, and physical resources. Enthusiastic work engagement depends on not only individuals’ competence for work but also the support of internal and external environmental resources perceived by individuals, including: (1) whether organizational environments encourage and applaud the behaviors of work engagement; (2) whether individuals receive full support from leaders in case of setbacks and difficulties; and (3) whether work achievements are duly rewarded. Organizational ecology affects the resource recycling status of individuals in work, for example, loss of resources due to blind engagement, and resource balance or accumulation following their acquisition. According to the acting mechanism of resources, the profit and loss status or expectation of resources directly determines individuals’ motivations for work engagement. According to the conservation of resource theory, the behaviors of work engagement are motivated by the supply of external resources (e.g., normative and reasonable organizational environment, and unbiased promotion mechanism). In the absence of an initial advantageous environment, an overemphasis of identification with and devotion to work may lead to withdrawal behaviors in work, because people fear that they are not compensated for loss of resources.

In summary, the motivation dynamic of work engagement is a process of self-determination along with internal and external regulation, which is based on satisfying basic psychological needs. Reasonable allocation of external motivation resources facilitates the transformation of individual behaviors from external regulation to internal regulation and gradually strengthens the core role of self-determination in work engagement.

## Data Availability Statement

The raw data supporting the conclusions of this article will be made available by the authors, without undue reservation, to any qualified researcher.

## Ethics Statement

The studies involving human participants were reviewed and approved by the Chinese National Office for Philosophy and Social Science. The patients/participants provided their written informed consent to participate in this study.

## Author Contributions

XL conducted the research and wrote the manuscript.

## Conflict of Interest

The author declares that the research was conducted in the absence of any commercial or financial relationships that could be construed as a potential conflict of interest.
